# A Critical Appraisal of Reporting in Randomized Controlled Trials Investigating Osteopathic Manipulative Treatment: A Meta-Research Study

**DOI:** 10.3390/jcm13175181

**Published:** 2024-08-31

**Authors:** Gabriele Zambonin Mazzoleni, Andrea Bergna, Francesca Buffone, Andrea Sacchi, Serena Misseroni, Marco Tramontano, Fulvio Dal Farra

**Affiliations:** 1 SOMA—Istituto Osteopatia Milano, Viale Sarca 336 F, 20126 Milan, Italy; gzamboninmazzoleni@studenti.uninsubria.it (G.Z.M.); francescabuffone.ost@gmail.com (F.B.); andrea.sacchi97@hotmail.com (A.S.); serenamisseroni@icloud.com (S.M.); fulvio.dalfarra@unibs.it (F.D.F.); 2 Physiotherapy Degree Course, Department of Medicine and Technology Innovation, Università degli Studi dell’Insubria, 21100 Varese, Italy; 3 AISO—Associazione Italiana Scuole di Osteopatia, 65125 Pescara, Italy; 4 PPCR, Harvard T.H. Chan School of Public Health—ECPE, Boston, MA 02115-6096, USA; 5 Department of Biomedical and Neuromotor Sciences, University of Bologna, 40126 Bologna, Italy; marco.tramontano@unibo.it; 6 Unit of Occupational Medicine, IRCCS Azienda Ospedaliero-Universitaria di Bologna, 40138 Bologna, Italy; 7 Department Information Engineering, University of Brescia, Via Branze 38, 25123 Brescia, Italy

**Keywords:** meta-research, reporting, randomized controlled trial, checklist, research quality, RCT, osteopathic manipulative treatment, osteopathy

## Abstract

**Background/Objectives**: In osteopathy, it becomes necessary to produce high-quality evidence to demonstrate its effectiveness. The aim of this meta-research study is to assess the reporting quality of RCTs published in the osteopathic field. **Methods**: The protocol was preliminarily registered on the “Open Science Framework (OSF)” website. For reporting, we considered the PRISMA 2020 checklist. We included all the RCTs, published between 2011 and 2023, investigating the effectiveness of Osteopathic Manipulative Treatment (OMT) in any possible condition. The search process was conducted on four major biomedical databases including PubMed, Central, Scopus and Embase. A data extraction form was implemented to collect all relevant information. The completeness of reporting was calculated as the percentage of adherence to the CONSORT checklist; the Cochrane ROB 2 tool was considered to assess the risk of bias (RoB) in the following five major domains: randomization (D1), interventions (D2), missing data (D3), outcome measurement (D4), selective reporting (D5). **Results**: A total of 131 studies were included and the overall adherence was 57%, with the worst section being “other information” (42%). Studies with a lower RoB showed higher adherence to the CONSORT. The “results” section presented the highest differences as follows: D1 (−36.7%), D2 (−27.2%), D3 (−21.5%) and D5 (−25.5%). Significant correlations were also found between the preliminary protocol registration, higher journal quartile, publication in hybrid journals and the completeness of reporting (β: 19.22, CI: 14.45–24.00, *p* < 0.001; β: 5.41; CI: 2.80–8.02, *p* ≤ 0.001; β: 5.64, CI: 1.06–10.23, *p* = 0.016, respectively). **Conclusions**: The adherence to the CONSORT checklist in osteopathic RCTs is lacking. An association was found between a lower completeness of reporting and a higher RoB, a good journal ranking, publication in hybrid journals and a prospective protocol registration. Journals and authors should adopt all the strategies to adhere to reporting guidelines to guarantee generalization of the results arising from RCTs.

## 1. Introduction

The osteopathic profession is relatively new in the scientific panorama, and it has yet to be regulated as a healthcare profession in several countries [1,2]. To ensure this profession gains recognition in the healthcare field, it is necessary to produce high-quality research to demonstrate its efficacy in various clinical contexts [3]. To date, different systematic reviews about the effectiveness of the osteopathic manipulative treatment in various clinical conditions have been carried out, reporting conflicting results and an overall low-quality level of evidence [4,5,6,7].

In this context, randomized controlled trials (RCTs) are considered the best study design to demonstrate the efficacy of an intervention in medicine [8]. Every published research article must report its information precisely to enhance transparency, clarity and reproducibility [9]. Therefore, it is fundamental that methodological quality and reporting are performed optimally. Methodological quality refers to the rigor with which a study is designed and conducted, while quality of reporting pertains to how well the study’s methods and findings are described in the published report. Both are crucial for evaluating the overall reliability and reproducibility of the research [10]. To better evaluate these aspects, meta-research studies have been increasingly used in recent years, focusing on various aspects such as how research is performed, reported and evaluated [11,12,13]. The aim of scientific reports should be to inform the community of clinicians and researchers about the enrolled patient population, intervention protocols, results and the generalizability of the findings. If these details are missed, the study will be poorly reproducible and thus not applicable in clinical practice [9].

In addition, inadequate reporting of information may adversely affect the assessment of the risk of bias (RoB), which is a crucial component for making an in-depth critical appraisal of a scientific article [14]. In fact, assessing internal validity requires a thorough evaluation of the RoB, which depends on how the information is reported by the authors in the paper. In this context, the Consolidated Standards of Reporting Trials (CONSORT) checklist is a widely recognized tool aimed at improving the transparency and completeness of reporting in RCTs [15]. Originally developed in 1996 and updated in 2010, the CONSORT checklist comprises a set of guidelines for authors to ensure clear and accurate reporting of key elements [9]. It consists of six main sections, each structured into different items. This checklist is supposed to be adopted by all authors of RCTs, and readers should use it to assess the completeness of article reporting.

Previous research has already highlighted the importance of adhering to reporting guidelines, thus improving the quality of research reporting [9,15]. However, different studies pointed out that many authors fail to follow CONSORT guidelines adequately [11,16]. To our knowledge, no systematic assessment of the reporting has been carried out in the context of osteopathy. The lack of systematic evaluations of reporting in the osteopathic field poses a significant challenge, as it limits the interpretability of research findings and constrains the advancement of evidence-based practice. Our study addresses this gap by offering a comprehensive evaluation of reporting quality in osteopathic RCTs, a crucial step toward enhancing research standards in this field.

Therefore, the primary aim of this meta-research study is to evaluate the adherence of randomized controlled trials investigating the effectiveness of osteopathic manipulative treatment to the CONSORT 2010 checklist, and to investigate any possible relationship with RoB, assessed using the Cochrane risk-of-bias tool 2.0 (RoB 2 tool). The secondary aim is to investigate whether there is a correlation between the completeness of reporting and other characteristics, such as year of publication, prospective protocol registration, journal quartile and publication options.

## 2. Materials and Methods

This is a meta-research study investigating the reporting quality level of RCTs belonging to the osteopathic field. Since there is no specific register for meta-research studies, we published the protocol on the OSF website (https://doi.org/10.17605/OSF.IO/QKBGU). For reporting, we considered the Preferred Reporting Items for Systematic Reviews and Meta-Analyses (PRISMA) 2020 checklist. The PRISMA checklist has been conceptualized as a checklist for systematic reviews [17]. Since there is no specific reporting checklist for meta-research studies, we implemented this tool as it is considered the most suitable for this study design [16]; subsequently, its use was adapted to suit the needs of our study.

### 2.1. Eligibility

We included all the RCTs with a parallel-groups design, published between January 2011 and December 2023, investigating the effectiveness of the osteopathic manipulative treatment in any possible medical condition, or in healthy people. Other study designs such as observational cohort or case-control studies, quasi-randomized and quasi-experimental clinical trials, single-subject design studies, subgroup and secondary analyses, editorials, commentaries and letters to the editor were excluded. The RCT protocols considered were on the clinicaltrials.gov website and their reporting was assessed only for the applicable items. We also included trials in which specific osteopathic modalities were applied (e.g., myofascial release, visceral manipulation, cranio-sacral treatment) only in the case the operators had a specific certification in those approaches. Abstracts, conference proceedings or RCTs written in languages other than English were consequently excluded.

### 2.2. Study Selection

A search strategy was developed by two blinded reviewers, reaching a final consensus with a third expert one. We considered “Mesh” and “Free-terms” such as “osteopathic manipulative treatment”, “osteopathy”, “osteopathic manipulation”, “myofascial release”, “cranio-sacral” and “visceral manipulation”, and we combined them considering their variations, according to the different databases modalities. 

**Figure 1:**
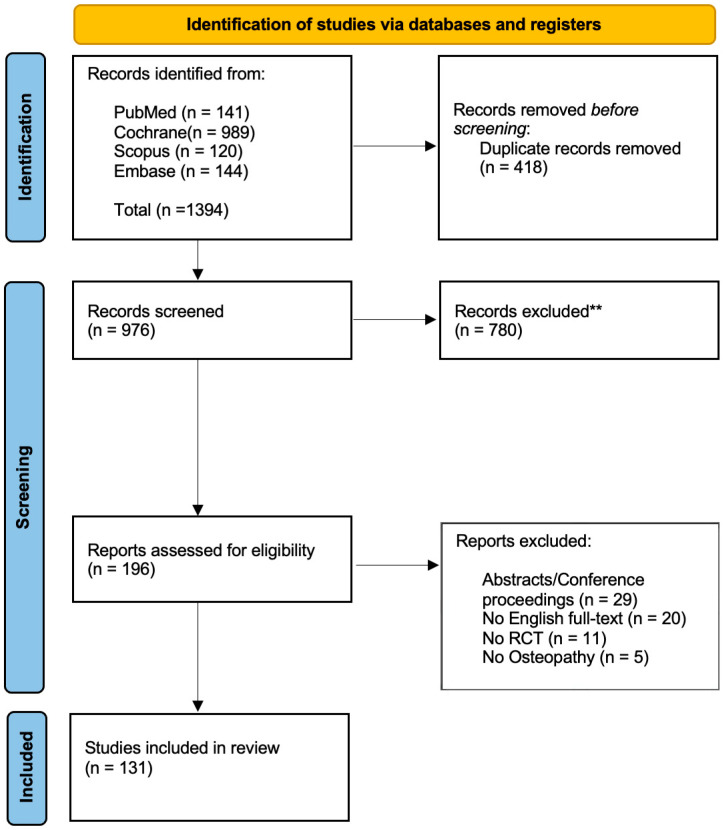
Flow diagram based on the PRISMA statement. ** = excluded after title/abstract screening.

The search process was conducted on four major biomedical databases including PubMed (MEDLINE), Central (Cochrane), Scopus and Embase. The study selection stage was performed through the web-app “Rayyan” [18]. Two blinded reviewers (GZM, FB) independently assessed the records based on the selection criteria. The first screening was performed on the title and abstract and, when necessary, by full-text reading. The two reviewers were blinded to each other’s assessments during the initial screening and full-text evaluation stages to minimize selection bias. A third reviewer (FDF) was recruited to resolve any potential disagreement. The selection process is detailed in Figure 1.

### 2.3. Data Collection Process

A data extraction form was conceived by all authors involved in this research and the following data were collected: first author’s name, country, completeness of reporting, risk of bias, publication year, journal characteristics (open access vs. hybrid), protocol registration (yes/no). Data were retrieved by the same two reviewers (GZM, FB) and any discrepancy was discussed with a third one (FDF). Before starting the extraction procedure, a total of 6 h of consensus training was implemented to guarantee uniformity in the extraction procedure.

### 2.4. Evaluating the Completeness of Reporting

The completeness of reporting was assessed by the same two blinded reviewers (with a third one used in case of conflict), and it was calculated as the adherence to each of the 25 items in the CONSORT checklist [15]. Each item was rated as 1 if it was well described, 0 if the information was missing, and 2 if the item was not applicable. An item was classified as not applicable when the authors did not provide a description for it and the absence of such information was justified.

The adherence to the checklist was calculated considering the relationship between the described items and the items applicable, thus ranging from 0% (no adherence) to 100% (maximum adherence). All requested information present in the checklist were retrieved in each study, without requesting any additional information from the authors.

### 2.5. Evaluating the Risk of Bias

To assess the RoB, the Cochrane RoB 2 tool was considered [19]. This tool consists of five different domains, each assessing the level of the risk (high, low, or some concerns). The five domains are as follows: randomisation process (D1); deviations from intended interventions (D2); missing outcome data (D3); outcome measurement (D4); selection of the reported results (D5). The RoB was assessed by the same two blinded reviewers, with a third one consulted in case of discrepancies.

### 2.6. Statistical Analysis

The overall adherence to the CONSORT checklist was calculated as a percentage, representing the total number of described items divided by the total number of applicable items. The adherence to each item and section of the checklist across all studies was determined by calculating the percentage of times each item was described and reported in relation to the total number of studies in which the item was applicable. To assess if there is a relationship between the completeness of reporting and the RoB, a multivariable linear regression analysis was implemented. The dependent variable was the overall adherence for each study; the independent variables were each domain and the overall RoB.

The potential relationship between the overall adherence to the CONSORT checklist and other characteristics such as publication year, journal ranking (quartile range), publication options and study protocol was investigated through a multivariable linear regression analysis, with the overall adherence as a dependent variable, and the other characteristics of the studies as independent ones.

The analysis was conducted by using the software IBM SPSS Statistics v.24.0.

## 3. Results

### 3.1. Adherence to CONSORT Checklist (Completeness of Reporting)

Of the 196 records assessed for eligibility, a total of 131 studies were included in the analysis; 29 were excluded since they corresponded to congress abstracts or conference proceeding, 11 presented issues in the randomization process (e.g., quasi-randomized, cross-over design), 5 were carried out by non-osteopath practitioners and the remaining were not written in English (Figure 1).

The mean overall adherence to the CONSORT checklist was 57%, though a great variability across the studies was detected (18−97%).

**Figure 2:**
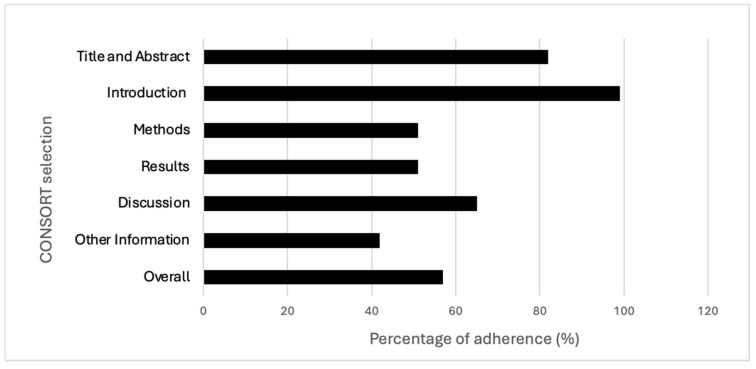
Adherence across CONSORT sections and overall adherence.

**Figure 3:**
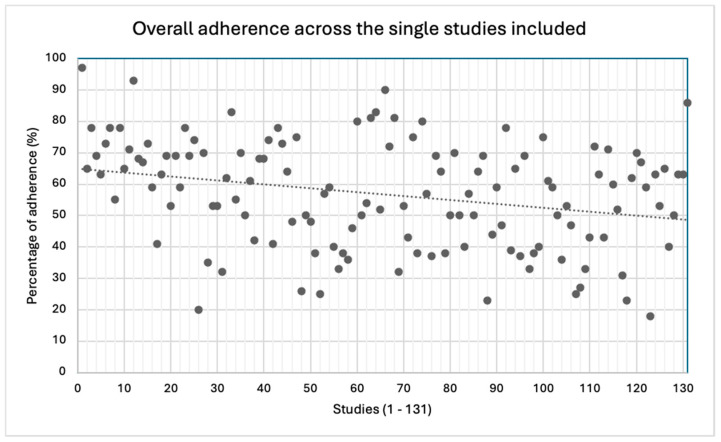
Overall adherence across the single studies included.

The best-described section in the checklist was the introduction (99%), and the worst was the one related to “other information” (42%). The items showing the best results in terms of adherence (100%) were the ones regarding “specific objectives or hypotheses” (2b) and “explanation of any interim analyses and stopping guidelines” (7b); conversely, the worst (4%) was the one checking for the presence of a detailed source reporting the study protocol (24). Items 3b, 6b, 7b, 12b, 14b and 18 were not applicable in most of the RCTs, and this may have influenced the overall results. Further details are reported in Table 1 and Figure 2 and Figure 3. Additional information regarding characteristics and references of the included studies are reported in Supplementary Tables S1 and S2.

### 3.2. Relationship between Completeness of Reporting and Risk of Bias

The risk of bias (RoB) was assessed using the Cochrane RoB 2.0 tool, focusing on the following five domains: randomization process (D1), deviations from intended interventions (D2), missing outcome data (D3), measurement of the outcome (D4) and selection of the reported results (D5). Detailed results are provided in Table 2, highlighting significant differences in reporting quality between studies with low and high RoB. Overall, studies with a lower RoB showed higher adherence to the CONSORT checklist. The first domain (D1) had the greatest differences in reporting in favour of the lower RoB studies, particularly regarding title and abstract (−20.1%), methods (−27.9%), results (−36.7%), other information (−22.5%) and overall adherence (−24.5%). The section of results presented the highest differences in terms of reporting between high- and low-risk studies, especially in D1 (−36.7%), D2 (−27.2%), D3 (−21.5%) and D5 (−25.5%).

The multivariable linear regression analysis (Table 3) showed how a lower risk in D1, D2, D3 and D5 was significantly associated with a better completeness of reporting. In particular, studies with a lower RoB in D1 and D5 showed beta unstandardized values (β) of 12.3 (8.9–15.8), *p* < 0.001, and 10.8 (7.2–14.4), *p* < 0.001, respectively. This finding means that studies with a higher RoB in D1 have an overall adherence to the CONSORT checklist 12.3% lower than those with “some concerns”, and 12.3% lower than those with a low RoB. In the same way, studies with a higher RoB in D5 have an overall adherence that is 21.6% lower than those with a low RoB. For the overall RoB, the trend is opposite (β = −9,4 CI: −15.15–3.838), *p* = 0.001.

### 3.3. Relationship between Completeness of Reporting and Protocol Registration, Journals Characteristics and Date of Publication

As an additional analysis, studies which published a preliminary protocol presented a higher adherence to the CONSORT checklist than studies which did not (β: 19.22, CI: 14.45–24.00, *p* < 0.001). Significant correlations were also found between the “higher journal quartile”, “publication options” and the “completeness of reporting”. Publishing in higher quartile journals showed a higher adherence to the checklist (β: 5.41; CI: 2.80–8.02, *p* ≤ 0.001). The articles published in hybrid journals had a higher adherence than articles published in open-access journals (β: 5.64, CI: 1.06–10.23, *p* = 0.016). No significant relationship between “year of publication” and “completeness of reporting” was found. Further details are reported in Table 4 and specific information on the journal characteristics are provided in Supplementary Table S3.

## 4. Discussion

### 4.1. Summary of Results

The primary aim of this meta-research study was to investigate the completeness of reporting in osteopathic research by associating it to the methodological quality of the studies. Our results provide crucial insights into understanding the current state of the research in osteopathy; this aspect seems to be paramount considering the importance of evidence-based practice in the healthcare profession [20].

Our findings suggest that the RCTs’ overall level of adherence to the CONSORT checklist is critical. Indeed, the percentages of adherence, both aggregate and divided according to each section, appeared mostly unsatisfactory.

In detail, more than one of two checklist items had information (51%) that was not adequately reported in the “methods” section. In other words, about half of the information requested by the CONSORT checklist is missing in the osteopathic RCTs methods section, preventing readers from fully comprehending the applied methodologies and identifying possible sources of bias. Similarly, the same percentage of adherence (51%) has been observed with regards to the “results” section, suggesting a lower reporting of important data, especially participants’ baseline characteristics, effects size with their precision, and harms or adverse events. Missing information related to the results presentation corresponds to a specific risk of bias (outcome reporting bias) [21]; as such, it may threaten the internal validity of the study [22].

This trend was also confirmed regarding the discussion section, where the percentage of adherence was slightly higher, reaching 65% of the items adequately reported. By analysing each item in the checklist, the vast majority of the issues were found to be related to generalizability (item 21), where only 21% of the studies provided a discussion of the external validity and the applicability of the obtained findings. As known, guaranteeing the generalizability of the study findings represents a crucial aspect in research [23]. In addition, although the section “other information” showed a critical adherence as a vast proportion of RCTs did not report either the number of registrations of the study or the availability of the protocol. As reported by several authors, to preliminarily register the protocol of a clinical study and have it available are fundamental aspects for the transparency and trustworthiness of the study [24,25,26]. Conversely, better levels of adherence were observed both for the “introduction” and “title and abstract” sections (99% and 82%, respectively).

Our analysis also correlated the RCTs’ levels of reporting adherence to the presence of risk of bias, obtaining results worthy of discussion. As expected, studies showing a higher risk of bias seem to have lower adherence to the CONSORT checklist. This fact appears to be particularly evident considering biases arising from the randomization process (D1) and the overall adherence to the checklist, where almost a 25% difference has been observed in favour of low RoB studies. In addition, data showed how RCTs with issues in the randomization phase (D1) tend to have a lower reporting in the methods and results section, and that trials characterised by a higher risk of bias in deviations from intended interventions (D2), missing outcome data (D3) and in the selection of the reported results (D5) have a lower adherence, mostly in the results section.

All these descriptive data have been confirmed by our multivariate analysis, which showed how a higher risk of bias in the randomization process, deviation from intended procedures, missing outcome data and selectiveness of the reported results seems to strongly predict lower levels of reporting.

Not surprisingly, a lower overall RoB was not associated with better reporting. This aspect can be explained by the modality of the Cochrane 2.0 RoB tool, in which the presence of a single suspected bias heavily influences the overall judgement. For this reason, we consider the association for each single domain more indicative to explain the association between reporting and RoB.

In the same way, a higher journal quartile, the open access publication option and the availability of a preliminary protocol registration, though not the year of publication, are associated with better reporting.

The results we obtained appear to be consistent with those coming from other similar reviews, which investigated the reporting of non-pharmacological trials. Specifically, a scoping review published in 2023 found overall incomplete intervention reporting in 113 trials dealing with manual therapy applied by different professionals (physiotherapists, chiropractor and osteopaths) for neck pain [27]. In the same way, another recent review noticed how reporting in manual therapy RCTs is not improving over time, then stressed the importance of an extensive systematic use of the reporting checklists [28]. In addition, other authors specifically focused on the rehabilitation field, highlighting the need for better reporting as it is currently suboptimal [16,29,30]. In a different context, Candy and colleagues retrieved the same trend in educational and psychotherapeutic clinical trials [31].

This similar trend directly leads to the need for standardization and generalization in this field. Actually, the measurability of an intervention depends on the quality of reporting of the intervention itself [32]. For this purpose, specific tools had been developed and adopted in research, such as the “Template for Intervention Description and Replication (TIDieR) [33,34], a 12-item checklist useful to describe the intervention in detail. Regarding this topic, Alvarez and colleagues suggest the use of TIDieR in the field of manual and manipulative therapies, intrinsically affected by higher levels of variability according to operator attitudes. In this sense, its usage might help in ensuring both internal and external validity of a clinical trial [35].

### 4.2. Implications for Future Research

As stated above, this first meta-research study in the osteopathic field may represent a landmark to address future research in this context. Although the current edition of the CONSORT checklist has been available since 2010, the overall adherence of RCTs is not considered satisfactory. Explanations could involve the fact that many journal editors might not consider the adherence to checklist as necessary to conduct the peer-review process and consequently, for publishing, a similar argument may cover the reviewers themselves. Alternatively, although the authors of the trials declare the use of the CONSORT, they could not follow it properly.

From a different perspective, a lack of reporting could also hide some relevant methodological issues in the research process [36]. Actually, the absence of relevant information in the text of the article may be interpreted as a missing methodological step in the research. Examples of missing methodological steps are as follows: blinding and randomization procedures, outcome measurements and protocol registration may have been skipped, or at least, not properly performed. Furthermore, the inherent nature of manual therapies often makes blinding of practitioners challenging, if not impossible. This limitation impacts both the internal validity of trials and its relationship with the adherence to CONSORT guidelines. To our knowledge, only a few studies addressed this issue with a blinding questionnaire. Future studies should consider these limitations, exploring alternative strategies to reduce the RoB.

Possible solutions can vary. All indexed journals should require a reporting guidelines checklist as a mandatory step to start the peer-review process and, not least, automatic tools might be implemented to check the effective adherence [37]. Regarding this step, the use of artificial intelligence could represent an option, considering the initial promising results [38]. In this context, the involvement of the reviewers in their work is arguable, and a policy of incentives can be taken into consideration to guarantee a high-quality peer-review process. 

On the other hand, the authors should pay close attention to follow reporting guidelines. They should systematically submit the checklist alongside the manuscript, detailing it with the utmost precision. This could help in providing maximum readability and transparency of their research works. 

Our findings might have important implications both for clinical practice and future research in osteopathy. Clinicians should be aware of the importance of reporting when they read and interpret RCT results; particular attention should be given to the methods section, where critical aspects are present. Researchers in the osteopathic field should consider the relevance of complete and adequate reporting to allow interpretability and generalization of their study findings. Finally, further meta-research investigations in osteopathy should be implemented in the near future. Alternative and complementary medicines are relatively new in the research panorama; thus, a mapping of the main methodological issues could be helpful to provide specific “calls to action”. Possible themes requiring attention might be the detailing of the manipulative techniques in the protocols, and the adherence of the systematic review to the PRISMA checklist [39].

### 4.3. Limitations

We acknowledge several limitations that may affect the interpretation of our results. Firstly, there are intrinsic limitations related to the nature of meta-research, as it is a relatively new typology of study design, and specific methodological guidelines are still missing. Furthermore, the CONSORT checklist was designed to aid in the standardization and reproducibility of RCTs, rather than to assess the quality of reporting. Related to this aspect, there are currently no specific tools available in the literature on how to provide a qualitative judgement of the reporting. To mitigate this issue, the assessors underwent six hours of consensus training and subsequently carried out a trial with ten randomly selected articles from those included in the study, obtaining a full inter-operator agreement.

Secondly, to the best of our knowledge, no specific reporting guidelines currently exist for meta-research studies. Therefore, we adhered to the PRISMA checklist, as systematic reviews appear as the closest type of research.

## 5. Conclusions

The adherence to the CONSORT checklist in osteopathic RCTs is lacking overall, and the section with the lowest adherence appeared to be “methods and results”. An association was found between a lower completeness of reporting and a higher risk of bias, considering each single domain of the Cochrane RoB 2.0 toll. In addition, a good journal ranking, publications on hybrid journals and a prospective protocol registration were related to better reporting. Journals and authors should adopt all the necessary strategies to adhere to reporting guidelines. Further meta-research studies could be strategic to guide future research in osteopathy, to allow generalization of the results.

## Figures and Tables

**Table 1:** Mean adherence across each item of the CONSORT checklist in RCTs published in the osteopathic field.

		CONSORT Items	Studies Where Item Was Not Applicable (*n*)	Mean Adherence (%) Calculated in Studies Where Item Was Applicable
**Title and Abstract**	1a	Identification as a randomised trial in the title	0	65
	1b	Structured summary of trial design, methods, results, and conclusions	0	99
**Introduction** Background and objectives	2a	Scientific background and explanation of rationale	0	98
	2b	Specific objectives or hypotheses	0	100
**Methods** Trial design	3a	Description of trial design including allocation ratio	0	24
	3b	Important changes to methods after trial commencement (such as eligibility criteria), with reasons	128	67
Participants	4a	Eligibility criteria for participants	0	95
	4b	Settings and locations where the data were collected	0	50
Interventions	5	The interventions for each group with sufficient details to allow replication, including how and when they were actually administered	0	79
Outcomes	6a	Completely defined prespecified primary and secondary outcome measures, including how and when they were assessed	29	87
	6b	Any changes to trial outcomes after the trial commenced, with reasons	129	50
Sample size	7a	How sample size was determined	29	57
	7b	When applicable, explanation of any interim analyses and stopping guidelines	127	100
Sequence generation	8a	Method used to generate the random allocation sequence	0	73
	8b	Type of randomization; details of any restriction (such as blocking and block size)	0	30
Allocation concealment	9	Mechanism used to implement the random allocation sequence, describing any steps taken to conceal the sequence until interventions were assigned	0	18
Implementation	10	Who generated the random allocation sequence, who enrolled participants, and who assigned participants to interventions	0	10
Blinding	11a	If performed, who was blinded after assignment to interventions and how	27	74
	11b	If relevant, description of the similarity of interventions	64	55
Statistical methods	12a	Statistical methods used to compare groups for primary and secondary outcomes	0	24
	12b	Methods for additional analyses, such as subgroup analyses and adjusted analyses	115	69
**Results** Participant flow	13a	For each group, the numbers of participants who were randomly assigned, received intended treatment, and were analysed for the primary outcome	0	69
	13b	For each group, losses and exclusions after randomization, together with reasons	45	53
Recruitment	14a	Dates defining the periods of recruitment and follow-up	0	40
	14b	Why the trial ended or was stopped	129	50
Baseline data	15	A table showing baseline demographic and clinical characteristics for each group	0	79
Numbers analysed	16	For each group, number of participants (denominator) included in each analysis and whether the analysis was by original assigned groups	0	24
Outcomes and estimation	17a	For each primary and secondary outcome, results for each group, and the estimated effect size and its precision (such as 95% CI)	0	40
	17b	For binary outcomes, presentation of both absolute and relative effect sizes is recommended	124	71
Ancillary analyses	18	Results of any other analyses performed, including subgroup analyses and adjusted analyses, distinguishing prespecified from exploratory	116	73
Harms	19	All important harms or unintended effects in each group	0	47
**Discussion** Limitations	20	Trial limitations, addressing sources of potential bias, imprecision, and, if relevant, multiplicity of analyses	0	85
Generalizability	21	Generalizability (external validity, applicability) of the trial findings	0	21
Interpretation	22	Interpretation consistent with results, balancing benefits and harms, and considering other relevant evidence	0	91
**Other information** Registration	23	Registration number and name of trial registry	0	50
Protocol	24	Where the full trial protocol can be accessed, if available	0	4
Funding	25	Sources of funding and other support (such as supply of drugs), role of funders	0	73

In bold the name of the “main sections of the CONSORT checklist”.

**Table 2:** Differences in reporting between studies with low risk of bias and high risk of bias, for each domain of the ROB 2 tool and each section of the CONSORT checklist.

	L	H	Diff	L	H	Diff	L	H	Diff	L	H	Diff	L	H	Diff
**Title and Abstract**	91.1	71.0	−20.1	95.1	78.7	−16.4	83.1	76.9	−6.2	82.6	78.5	−4.1	89.8	78.1	−11.7
**Introduction**	98.0	100	2.0	98.3	98.9	0.6	98.6	100	1.4	98.3	100	1.7	98.3	100	1.7
**Methods**	63.8	35.9	−27.9	64.6	50.7	−13.9	51.4	43.4	−8	53.0	43.0	−10.0	60.8	51.1	−9.7
**Results**	69.6	32.9	−36.7	76.8	49.6	−27.2	51.8	30.3	−21.5	50.3	56.4	6.1	68.2	42.7	−25.5
**Discussion**	70.0	59.1	−10.9	79.1	62.5	−16.6	65.5	61.0	−4.5	66.9	56.7	−10.2	70.7	61.9	−8.8
**Other information**	53.7	31.2	−22.5	51.1	42.8	−8.3	42.3	43.1	0.8	43.4	37.7	−5.7	62.1	49.5	−12.6
**Overall adherence**	69.1	44.6	−24.5	72.0	56.4	−15.6	57.7	48.4	−9.3	58.0	54.2	−3.8	68.4	55.6	−12.8

Abbreviations. L: low; H: high; Diff: difference.

**Table 3:** Multivariable linear regression analysis with domains and overall risk of bias as independent variables, and overall adherence to the CONSORT checklist as dependent variable.

Coefficients				
			95% CI	
Variables	Beta Unstandardized (β)	*p*	Lower	Upper
D1	12.368 *	<0.001 **	8.901 *	15.835 *
D2	7.218 *	0.001 **	2.848 *	11.589 *
D3	4.007 *	0.044 *	0.117 *	7.898 *
D4	0.798	0.650	−2.671	4.266
D5	10.836 *	<0.001 **	7.267 *	14.405 *
Overall RoB	−9.494	0.001	−15.15	−3.838

Abbreviations. D1: RoB arising from the randomization process; D2: RoB because of deviations from the intended interventions; D3: RoB because of missing outcome data; D4: RoB in measurement of the outcome; D5: RoB in selection of the reported result; *: *p* < 0.05; **: *p* < 0.001.

**Table 4:** Multivariable linear regression analysis with “year of publication”, “journal ranking”, “publication options” and “preliminary protocol registration” as independent variables, and overall adherence to the CONSORT checklist as dependent variable.

Coefficients				
			95% CI	
Variables	Beta Unstandardized (β)	*p*	Lower	Upper
Recent year of publication	0.453	0.168	−0.193	1.098
Higher quartile	5.416 *	<0.001 **	2.805 *	8.026 *
Open Access publication	−5.649 *	0.016 *	−10.231 *	−1.067 *
Preliminary protocol	19.226 *	<0.001 **	14.452 *	24.001 *

Abbreviations. *: *p* < 0.05; **: *p* < 0.001.

## Data Availability

Available upon request to the corresponding author.

## Supplementary Materials

The following supporting information can be downloaded at: https://www.mdpi.com/article/10.3390/jcm13175181/s1, Table S1: characteristics of the included studies; Table S2: references of the included studies; Table S3: characteristics of the journals included.
