# Supplementary material for: A Critical Appraisal of Reporting in Randomized Controlled Trials Investigating Osteopathic Manipulative Treatment: A Meta-Research Study
Source: J Clin Med. 2024 Aug 31;13(17):5181. doi: 10.3390/jcm13175181 (PMC11396362; doi:10.3390/jcm13175181)
Supplement: Supplementary file 1 [file jcm-13-05181-s001.zip › jcm-3176682-supplementary.pdf]

Supplementary Table S1. Characteristics of the included studies.

Registered Protocol	(n)	Percentage (%)
Yes	66	50,38
No	65	49,61
**Publication Option**		
Hybrid	33	52,38
Open access	30	47,61
**Publication Year**		
2011	8	6,10
2012	6	4,58
2013	11	8,39
2014	11	8,39
2015	7	5,34
2016	3	2,29
2017	5	3,81
2018	12	9,16
2019	11	8,39
2020	21	16,03
2021	14	10,68
2022	16	12,21
2023	6	4,58
**Journals**		
Journal of Osteopathic Medicine	24	18,32%
Journal of Bodywork & Movement Therapies	15	11,45%
Complementary therapies in medicine	6	4,58%
International Journal of Osteopathic Medicine	6	4,58%
The Journal of Alternative and Complementary Medicine	5	3,82%
Healthcare	4	3,05%
PLOS ONE	4	3,05%
Evidence-Based Complementary and Alternative Medicine	3	2,29%
Scientific Reports	3	2,29%
International Journal of Environmental Research and Public Health	2	1,53%
Complementary Therapies in Clinical Practice	2	1,53%
Anaesthesia, Pain & Intensive Care	2	1,53%
Journal of Manipulative and Physiological Therapeutics	2	1,53%
Archives of Disease in Childhood	2	1,53%
Journal of Clinical Medicine	2	1,53%
Journal of Back and Musculoskeletal Rehabilitation	2	1,53%
BMC Complementary and Alternative Medicine	2	1,53%
Therapeutic Advances in Musculoskeletal Disease	2	1,53%
Clinical Rehabilitation	1	0,76%
Journal of Men's Health	1	0,76%
The Annals of Clinical and Analytical Medicine	1	0,76%

European Journal of Obstetrics & Gynecology and Reproductive Biology	1	0,76%
Frontiers in Behavioral Neuroscience	1	0,76%
NeuroRehabilitation	1	0,76%
American Journal of Perinatology	1	0,76%
Annals of Family Medicine	1	0,76%
Manual Therapy	1	0,76%
Medicine	1	0,76%
Spinal Cord	1	0,76%
Ortopedia Traumatologia Rehabilitacja	1	0,76%
Experimental and Therapeutic Medicine	1	0,76%
Diseases of the Esophagus	1	0,76%
Alternative Therapies in Health and Medicine	1	0,76%
Disability and Rehabilitation	1	0,76%
American Journal of Obstetrics and Gynecology	1	0,76%
Journal of Human Lactation	1	0,76%
The American Academy of Osteopathy Journal	1	0,76%
Nursing Practice Today	1	0,76%
Rheumatology International	1	0,76%
The American Journal of Cardiology	1	0,76%
Giornale Italiano di Ostetricia e Ginecologia	1	0,76%
Cureus	1	0,76%
Gazzetta Medica Italiana Archivio per le Scienze Mediche	1	0,76%
Behavioural Sciences	1	0,76%
Complementary Medicine Research	1	0,76%
Turkish Society of Physical Medicine and Rehabilitation	1	0,76%
Journal of Chiropractic Medicine	1	0,76%
Life	1	0,76%
Journal of Complementary and Integrative Medicine	1	0,76%
BMC Pediatrics	1	0,76%
Journal of Manual and Manipulative Therapy	1	0,76%
Acta Obstetricia et Gynecologica Scandinavica	1	0,76%
The Clinical Journal of Pain	1	0,76%
Archives of Physical Medicine and Rehabilitation	1	0,76%
JAMA Internal Medicine	1	0,76%
Clinical Cases in Mineral and Bone Metabolism	1	0,76%
The Annals Of Thoracic Surgery	1	0,76%
CED Clinical and Experimental Dermatology	1	0,76%
Frontiers in Physiology	1	0,76%
Brain Sciences	1	0,76%
Journal of the Pakistan Medical Association	1	0,76%
**Country**		
Italy	35	26,72
USA	23	17,56
Spain	13	9,92
Germany	13	9,92
Brazil	10	7,63
France	7	5,34
Poland	6	4,58

Belgium	4	3,05
England	3	2,29
Egypt	3	2,29
Iran	3	2,29
Austria	3	2,29
Canada	2	1,53
Turkey	2	1,53
Portugal	1	0,76
Pakistan	1	0,76
New Zealand	1	0,76
Sweden	1	0,76

Supplementary Table S2. References of the studies included.

	ID	DOI	Authors	Title	Citation
Nguyen C 2021	1	10.1001/jamainternmed.2021.0005	Nguyen C, Boutron I, Zegarra-Parodi R, Baron G, Alami S, Sanchez K, Daste C, Boisson M, Fabre L, Krief P, Krief G, Lefèvre-Colau MM, Rannou F.	Effect of Osteopathic Manipulative Treatment vs Sham Treatment on Activity Limitations in Patients With Nonspecific Subacute and Chronic Low Back Pain. A Randomized Clinical Trial	JAMA Intern Med. 2021 May 1;181(5):620-630. doi: 10.1001/jamainternmed.2021.0005. PMID: 33720272; PMCID: PMC7961471.
Eguaras N 2019	2	10.3390/jcm8101738	Nuria Eguaras, Elena Sonsoles Rodríguez-López, Olga Lopez-Dicastillo, M. Ángeles Franco-Sierra, François Ricard, Ángel Oliva-Pascual-Vaca.	Effects of Osteopathic Visceral Treatment in Patients with Gastroesophageal Reflux: A Randomized Controlled Trial	J Clin Med. 2019 Oct 19;8(10):1738. doi: 10.3390/jcm8101738. PMID: 31635110; PMCID: PMC6832476.
Schwerla F 2020	3	10.1016/j.jbmt.2020.02.009	Schwerla F, Hinse T, Klosterkamp M, Schmitt T, Rütz M, Resch KL.	Osteopathic treatment of patients with shoulder pain. A pragmatic randomized controlled trial	J Bodyw Mov Ther. 2020 Jul;24(3):21-28. doi: 10.1016/j.jbmt.2020.02.009. Epub 2020 Feb 22. PMID: 32825990.

Groisman S 2020	4	10.1016/j.jbmt.2019.11.002	Groisman S, Malysz T, de Souza da Silva L, Rocha Ribeiro Sanches T, Camargo Bragante K, Locatelli F, Pontel Vigolo C, Vaccari S, Homercher Rosa Francisco C, Monteiro Steigleder S, Jotz GP	Osteopathic manipulative treatment combined with exercise improves pain and disability in individuals with non-specific chronic neck pain: A pragmatic randomized controlled trial	J Bodyw Mov Ther. 2020 Apr;24(2):189-195. doi: 10.1016/j.jbmt.2019.11.002. Epub 2019 Nov 13. PMID: 32507144
Marti Salvador M 2018	5	10.1016/j.apmr.2018.04.022	Martí-Salvador M, Hidalgo-Moreno L, Doménech-Fernández J, Lisón JF, Arguisuelas MD	Osteopathic Manipulative Treatment Including Specific Diaphragm Techniques Improves Pain and Disability in Chronic Nonspecific Low Back Pain: A Randomized Trial	Arch Phys Med Rehabil. 2018 Sep;99(9):1720-1729. doi: 10.1016/j.apmr.2018.04.022. Epub 2018 May 19. PMID: 29787734.
Haller H 2016	6	10.1097/AJP.0000000000000290	Haller H, Lauche R, Cramer H, Rampp T, Saha FJ, Ostermann T, Dobos G.	Craniosacral Therapy for the Treatment of Chronic Neck Pain: A Randomized Sham-controlled Trial	Clin J Pain. 2016 May;32(5):441-9. doi: 10.1097/AJP.0000000000000290. PMID: 26340656; PMCID: PMC4894825.
Schwerla F 2015	7	10.7556/jaoa.2015.087	Schwerla F, Rother K, Rother D, Ruetz M, Resch KL.	Osteopathic Manipulative Therapy in Women With Postpartum Low Back Pain and Disability: A Pragmatic Randomized Controlled Trial	J Am Osteopath Assoc. 2015 Jul;115(7):416-25. doi: 10.7556/jaoa.2015.087. PMID: 26111129.
Brandl A 2021	8	10.3390/life12060868	Brandl A, Egner C, Schleip R.	Immediate Effects of Myofascial	Life 2021, 11, 845". Life (Basel). 2022 Jun 10;12(6):868. doi: 10.3390/life12060868.

			Reply to Kudus, A.L.	Release on the Thoracolumbar Fascia and Osteopathic Treatment for Acute Low Back Pain on Spine Shape Parameters: A Randomized, Placebo-Controlled Trial	PMID: 35743899; PMCID: PMC9225237.
Manzotti A 2020 A	9	10.1016/j.ctcp.2020.101116	Manzotti A, Cerritelli F, Lombardi E, La Rocca S, Chiera M, Galli M, Lista G	Effects of osteopathic treatment versus static touch on heart rate and oxygen saturation in premature babies: A randomized controlled trial	Complement Ther Clin Pract. 2020 May;39:101116. doi: 10.1016/j.ctcp.2020.101116. Epub 2020 Feb 8. PMID: 32379655.
Raith W 2016	10	10.1186/s12906-016-0984-5	Raith W, Marschik PB, Sommer C, Maurer-Fellbaum U, Amhofer C, Avian A, Löwenstein E, Soral S, Müller W, Einspieler C, Urlesberger B.	General Movements in preterm infants undergoing craniosacral therapy: a randomised controlled pilot-trial	BMC Complement Altern Med. 2016 Jan 13;16:12. doi: 10.1186/s12906-016-0984-5. PMID: 26758035; PMCID: PMC4710971.
Castejon Castejon M 2022	11	10.1016/j.ctim.2022.102885	Castejón-Castejón M, Murcia-González MA, Todri J, Lena O, Chillón-Martínez R.	Treatment of infant colic with craniosacral therapy. A randomized controlled trial	Complement Ther Med. 2022 Dec;71:102885. doi: 10.1016/j.ctim.2022.102885. Epub 2022 Sep 13. Erratum in: Complement Ther Med. 2022 Dec;71:102903. PMID: 36113708.
Cerritelli F 2015 A	12	10.1371/journal.pone.0127370	Cerritelli F, Pizzolorusso G, Renzetti C, Cozzolino V, D'Orazio M, Lupacchini M, Marinelli B, Accorsi A, Lucci C,	A Multicenter, Randomized, Controlled Trial of Osteopathic Manipulative Treatment on Preterms	PLoS One. 2015 May 14;10(5):e0127370. doi: 10.1371/journal.pone.0127370. PMID: 25974071; PMCID: PMC4431716.

			Lancellotti J, Ballabio S, Castelli C, Molteni D, Besana R, Tubaldi L, Perri FP, Fusilli P, D'Incecco C, Barlafante G		
Manzotti A 2022	13	10.3390/healthcare10050813	Manzotti A, Cerritelli F, Lombardi E, Monzani E, Savioli L, Esteves JE, Galli M, La Rocca S, Biasi P, Chiera M, Lista G.	Osteopathic Manipulative Treatment Regulates Autonomic Markers in Preterm Infants: A Randomized Clinical Trial	Healthcare (Basel). 2022 Apr 27;10(5):813. doi: 10.3390/healthcare10050813. PMID: 35627950; PMCID: PMC9141319.
Coste J 2021	14	10.1177/1759720X211009017	Coste J, Medkour T, Maigne JY, Pérez M, Laroche F, Perrot S.	Osteopathic medicine for fibromyalgia: a sham-controlled randomized clinical trial a sham-controlled randomized clinical trial	Ther Adv Musculoskelet Dis. 2021 Apr 16;13:1759720X211009017. doi: 10.1177/1759720X211009017. PMID: 33948127; PMCID: PMC8053754.
Racca V 2017	15	10.1016/j.athoracsur.2016.09.110	Racca V, Bordoni B, Castiglioni P, Modica M, Ferratini M.	Osteopathic Manipulative Treatment Improves Heart Surgery Outcomes: A Randomized Controlled Trial	Ann Thorac Surg. 2017 Jul;104(1):145-152. doi: 10.1016/j.athoracsur.2016.09.110. Epub 2017 Jan 18. PMID: 28109570.
Rotter G 2022	16	10.1111/ced.15340	Rotter G, Ahnert MW, Geue AV, Icke K, Binting S, Tissen-Diabaté T, Roll S, Ortiz M, Reinhold T, Kass B, Staab D, Pfab F, Willich SN, Brinkhaus B.	Acupuncture and osteopathic medicine for atopic dermatitis: a three-armed, randomized controlled explorative clinical trial	Clin Exp Dermatol. 2022 Dec;47(12):2166-2175. doi: 10.1111/ced.15340. Epub 2022 Oct 27. PMID: 35875898.

Altinbilek T 2018	17	10.5606/tftrd.2018.1384	Altınbilek T, Murat S, Yumuşakhuylu Y, İçağasıoğlu A.	Osteopathic manipulative treatment improves function and relieves pain in knee osteoarthritis: A single-blind, randomized-controlled trial	Turk J Phys Med Rehabil. 2018 Mar 9;64(2):114-120. doi: 10.5606/tftrd.2018.1384. PMID: 31453500; PMCID: PMC6657763.
Tramontano M 2020 A	18	10.3390/brainsci10120969	Tramontano M, Cerritelli F, Piras F, Spanò B, Tamburella F, Piras F, Caltagirone C, Gili T.	Brain Connectivity Changes after Osteopathic Manipulative Treatment: A Randomized Manual Placebo-Controlled Trial	Brain Sci. 2020 Dec 11;10(12):969. doi: 10.3390/brainsci10120969. PMID: 33322255; PMCID: PMC7764238.
Castejon Castejon M 2019	19	10.1016/j.ctim.2019.07.023	Castejón-Castejón M, Murcia-González MA, Martínez Gil JL, Todri J, Suárez Rancel M, Lena O, Chillón-Martínez R.	Effectiveness of craniosacral therapy in the treatment of infantile colic. A T randomized controlled trial	Complement Ther Med. 2019 Dec;47:102164. doi: 10.1016/j.ctim.2019.07.023. Epub 2019 Aug 13. PMID: 31780018.
Tamburella F 2019	20	10.3389/fphys.2019.00403	Tamburella F, Piras F, Piras F, Spanò B, Tramontano M, Gili T	Cerebral Perfusion Changes After Osteopathic Manipulative Treatment: A Randomized Manual Placebo-Controlled Trial	Front Physiol. 2019 Apr 5;10:403. doi: 10.3389/fphys.2019.00403. PMID: 31024346; PMCID: PMC6460882.
Elden H 2013	21	10.1111/aogs.12096	Elden H, Östgaard HC, Glantz A, Marciniak P, Linnér AC, Olsén MF.	Effects of craniosacral therapy as adjunct to standard treatment for pelvic girdle pain in pregnant women: a multicenter,	Acta Obstet Gynecol Scand. 2013 Jul;92(7):775-82. doi: 10.1111/aogs.12096. Epub 2013 Mar 4. PMID: 23369067.

				single blind, randomized controlled trial	
Amatuzzi F 2021	22	10.1016/j.jmpt.2021.06.003	Amatuzzi F, Gervazoni Balbuena de Lima AC, Da Silva ML, Cipriano GFB, Catai AM, Cahalin LP, Chiappa G, Cipriano G Jr.	Acute and Time-Course Effects of Osteopathic Manipulative Treatment on Vascular and Autonomic Function in Patients With Heart Failure: A Randomized Trial	J Manipulative Physiol Ther. 2021 Jul-Aug;44(6):455-466. doi: 10.1016/j.jmpt.2021.06.003. Epub 2021 Aug 26. PMID: 34456043.
Lynen A 2022	23	10.1016/j.jbmt.2021.09.017	Andreas Lynen, Meike Scho€mitz, Maik Vahle, Anne Ja€kel, Michaela Rutz, Florian Schwerla.	Osteopathic treatment in addition to standard care in patients with Gastroesophageal Reflux Disease (GERD) e A pragmatic randomized controlled trial	NA
Cruser dA 2012	24	10.1179/2042618611Y.0000000016	Cruser dA, Maurer D, Hensel K, Brown SK, White K, Stoll ST.	A randomized, controlled trial of osteopathic manipulative treatment for acute low back pain in active duty military personnel	J Man Manip Ther. 2012 Feb;20(1):5-15. doi: 10.1179/2042618611Y.0000000016. PMID: 23372389; PMCID: PMC3267441.
Luceno Maradones A 2021	25	10.3390/healthcare9040394	Luceño-Mardones A, Luceño-Rodríguez I, Rodríguez-López ES, Oliva-Pascual-Vaca J, Rosety I, Oliva-Pascual-Vaca Á.	Effects of Osteopathic T9–T10 Vertebral Manipulation in Tonsillitis: A Randomized Clinical Trial	Healthcare (Basel). 2021 Apr 1;9(4):394. doi: 10.3390/healthcare9040394. PMID: 33916061; PMCID: PMC8065872.
Papa L 2012	26	10.1186/1477-7525-3-78.	Papa L, Mandara A, Bottali M,	A randomized control trial on the effectiveness of	Clin Cases Miner Bone Metab. 2012 Sep;9(3):179-83. Epub 2012 Dec 20. PMID:

			Gulisano V, Orfei S.	osteopathic manipulative treatment in reducing pain and improving the quality of life in elderly patients affected by osteoporosis	23289034; PMCID: PMC3535995.
Arcanjo GN 2022	27	10.1016/j.jcm.2022.02.005	Arcanjo GN, Pires JLVR, Jacinto MEM, Colares JM, Belo LMC, Lima POP, Vilaça-Alves J.	Comparison of the Effect of Osteopathic Manipulations and Exercises on the Myoelectric Activity of the Pelvic Floor: A Randomized Controlled Trial	J Chiropr Med. 2022 Jun;21(2):97-107. doi: 10.1016/j.jcm.2022.02.005. Epub 2022 Apr 21. PMID: 35774632; PMCID: PMC9237590.
Niewiadomski C 2020	28	10.1016/j.ctim.2019.102278	Niewiadomski C, Bianco RJ, Arnoux PJ, Evin M.	Isometric osteopathic manipulation influences on cervical ranges of motion T and correlation with osteopathic palpatory diagnosis: A randomized trial	Complement Ther Med. 2020 Jan;48:102278. doi: 10.1016/j.ctim.2019.102278. Epub 2019 Dec 16. PMID: 31987245.
Albers J 2018	29	10.1159/000464343	Albers J, Jäkel A, Wellmann K, von Hehn U, Schmidt T.	Effectiveness of 2 Osteopathic Treatment Approaches on Pain, Pressure-Pain Threshold, and Disease Severity in Patients with Fibromyalgia: A Randomized Controlled Trial	Complement Med Res. 2018;25(2):122-128. doi: 10.1159/000464343. Epub 2017 Sep 12. PMID: 28892807.
Castro Sanchez AM 2011	30	10.1177/0269215510375909	Castro-Sánchez AM, Matarán-Peñarrocha GA, Sánchez-Labraca N, Quesada-	A randomized controlled trial investigating the effects of craniosacral therapy on pain and heart rate	Clin Rehabil. 2011 Jan;25(1):25-35. doi: 10.1177/0269215510375909. Epub 2010 Aug 11. PMID: 20702514.

			Rubio JM, Granero-Molina J, Moreno-Lorenzo C.	variability in fibromyalgia patients	
Voigt K 2011	31	10.1089/acm.2009.0673	Voigt K, Liebnitzky J, Burmeister U, Sihvonen-Riemenschneider H, Beck M, Voigt R, Bergmann A.	Efficacy of Osteopathic Manipulative Treatment of Female Patients with Migraine: Results of a Randomized Controlled Trial	J Altern Complement Med. 2011 Mar;17(3):225-30. doi: 10.1089/acm.2009.0673. Epub 2011 Mar 8. PMID: 21385086.
Haiden N 2015	32	10.1371/journal.pone.0123530	Haiden N, Pimpel B, Kreissl A, Jilma B, Berger A.	Does Visceral Osteopathic Treatment Accelerate Meconium Passage in Very Low Birth Weight Infants?- A Prospective Randomized Controlled Trial	PLoS One. 2015 Apr 15;10(4):e0123530. doi: 10.1371/journal.pone.0123530. Erratum in: PLoS One. 2017 Nov 2;12 (11):e0187784. PMID: 25875011; PMCID: PMC4398405.
Cerritelli F 2013 A	33	10.1186/1471-2431-13-65	Cerritelli F, Pizzolorusso G, Ciardelli F, La Mola E, Cozzolino V, Renzetti C, D'Incecco C, Fusilli P, Sabatino G, Barlafante G.	Effect of osteopathic manipulative treatment on length of stay in a population of preterm infants: a randomized controlled trial	BMC Pediatr. 2013 Apr 26;13:65. doi: 10.1186/1471-2431-13-65. PMID: 23622070; PMCID: PMC3648440.
Mancini JD 2023	34	10.1515/jom-2022-0085	Mancini JD, Angelo N, Abu-Sbaih R, Kooyman P, Yao S.	Concussion-related visual memory and reaction time impairment in college athletes improved after osteopathic manipulative medicine: a randomized clinical trial	J Osteopath Med. 2022 Sep 30;123(1):31-38. doi: 10.1515/jom-2022-0085. PMID: 36172719.
Mancini D 2019 A	35	10.1016/j.jmpt.2018.08.001	Mancini D, Cesari M, Lunghi C, Benigni AM,	Ultrasound Evaluation of Diaphragmatic Mobility and	J Manipulative Physiol Ther. 2019 Jan;42(1):47-54. doi: 10.1016/j.jmpt.2018.08.001.

			Antonelli Incalzi R, Scarlata S.	Contractility After Osteopathic Manipulative Techniques in Healthy Volunteers: A Prospective, Randomized, Double-Blinded Clinical Trial	Epub 2019 Apr 5. PMID: 30955907.
Thomaz SR 2018	36	10.1016/j.jbmt.2017.07.011	Thomaz SR, Teixeira FA, de Lima ACGB, Cipriano Júnior G, Formiga MF, Cahalin LP.	Osteopathic manual therapy in heart failure patients: A randomized clinical trial	J Bodyw Mov Ther. 2018 Apr;22(2):293-299. doi: 10.1016/j.jbmt.2017.07.011. Epub 2017 Jul 29. PMID: 29861222.
Benjamin JG 2020	37	10.1016/j.jbmt.2020.02.014	Benjamin JG, Moran RW, Plews DJ, Kilding AE, Barnett LE, Verhoeff WJ, Bacon CJ.	The effect of osteopathic manual therapy with breathing retraining on cardiac autonomic measures and breathing symptoms scores: A randomised wait-list controlled trial	J Bodyw Mov Ther. 2020 Jul;24(3):282-292. doi: 10.1016/j.jbmt.2020.02.014. Epub 2020 Feb 25. PMID: 32826001.
Seiler M 2020	38	10.1016/j.jbmt.2020.06.028	Seiler M, Vermeylen B, Poortmans B, Feipel V, Dugailly PM.	Effects of non-manipulative osteopathic management in addition to physical therapy and rehabilitation on clinical outcomes of ankylosing spondylitis patients: A preliminary randomized clinical trial	J Bodyw Mov Ther. 2020 Oct;24(4):51-56. doi: 10.1016/j.jbmt.2020.06.028. Epub 2020 Jul 11. PMID: 33218555.
Tamburella F 2022	39	10.3390/healthcare10020210	Tamburella F, Princi AA, Piermaria J,	Neurogenic Bowel Dysfunction	Healthcare (Basel). 2022 Jan 21;10(2):210. doi: 10.3390/healthcare10020210.

			Lorusso M, Scivoletto G, Masciullo M, Cardilli G, Argentieri P, Tramontano M.	Changes after Osteopathic Care in Individuals with Spinal Cord Injuries: A Preliminary Randomized Controlled Trial	PMID: 35206825; PMCID: PMC8871877.
Castro Sanchez AM 2016	40	10.1089/acm.2016.0068	Castro-Sánchez AM, Lara-Palomo IC, Matarán-Peñarrocha GA, Saavedra-Hernández M, Pérez-Mármol JM, Aguilar-Ferrándiz ME.	Benefits of Craniosacral Therapy in Patients with Chronic Low Back Pain: A Randomized Controlled Trial	J Altern Complement Med. 2016 Aug;22(8):650-7. doi: 10.1089/acm.2016.0068. Epub 2016 Jun 27. PMID: 27347698.
Roncada G 2020	41	10.1016/j.jbmt.2020.03.004	Roncada G.	Osteopathic treatment leads to significantly greater reductions in chronic thoracic pain after CABG surgery: A randomised controlled trial	J Bodyw Mov Ther. 2020 Jul;24(3):202-211. doi: 10.1016/j.jbmt.2020.03.004. Epub 2020 Mar 17. PMID: 32825989.
Rolle G 2014	42	10.7556/jaoa.2014.136	Rolle G, Tremolizzo L, Somalvico F, Ferrarese C, Bressan LC.	Pilot trial of osteopathic manipulative therapy for patients with frequent episodic tension-type headache	J Am Osteopath Assoc. 2014 Sep;114(9):678-85. doi: 10.7556/jaoa.2014.136. PMID: 25170037.
Rotter G 2020	43	10.1177/1759720X20979853	Rotter G, Fernholz I, Binting S, Keller T, Roll S, Kass B, Reinhold T, Willich SN, Schmidt A, Brinkhaus B.	The effect of osteopathic medicine on pain in musicians with nonspecific chronic neck pain: a randomized controlled trial	Ther Adv Musculoskelet Dis. 2020 Dec 10;12:1759720X20979853. doi: 10.1177/1759720X20979853. PMID: 33354233; PMCID: PMC7734566.

Pizzolorusso G 2014	44	10.1155/2014/243539	Pizzolorusso G, Cerritelli F, Accorsi A, Lucci C, Tubaldi L, Lancellotti J, Barlafante G, Renzetti C, D'Incecco C, Perri FP	The Effect of Optimally Timed Osteopathic Manipulative Treatment on Length of Hospital Stay in Moderate and Late Preterm Infants: Results from a RCT	Evid Based Complement Alternat Med. 2014;2014:243539. doi: 10.1155/2014/243539. Epub 2014 Nov 25. PMID: 25506381; PMCID: PMC4260368.
Wyatt K 2011	45	10.1136/adc.2010.199877	Wyatt K, Edwards V, Franck L, Britten N, Creanor S, Maddick A, Logan S	Cranial osteopathy for children with cerebral palsy: a randomised controlled trial	Arch Dis Child. 2011 Jun;96(6):505-12. doi: 10.1136/adc.2010.199877. Epub 2011 Feb 24. PMID: 21349889.
Whelan G 2018	46	10.1016/j.jbmt.2017.05.009	Whelan G, Johnston R, Millward C, Edwards DJ	The immediate effect of osteopathic cervical spine mobilization on median nerve mechanosensitivity: A triple-blind, randomized, placebo-controlled trial	J Bodyw Mov Ther. 2018 Apr;22(2):252-260. doi: 10.1016/j.jbmt.2017.05.009. Epub 2017 May 18. PMID: 29861216.
Martelli M 2014	47	10.1016/j.ctim.2014.01.009	Martelli M, Cardinali L, Barlafante G, Pizzolorusso G, Renzetti C, Cerritelli F	Do placebo effects associated with sham osteopathic procedure occur in newborns? Results of a randomized controlled trial	Complement Ther Med. 2014 Apr;22(2):197-202. doi: 10.1016/j.ctim.2014.01.009. Epub 2014 Feb 4. PMID: 24731889.
Thomas E 2022	48	10.1016/j.jbmt.2022.05.017	Thomas E, Petrucci M, Barretti M, Messina G, Cavallaro AR, Bianco A	Effects of osteopathic manipulative treatment of the pivots on lower limb function in young professional football players	J Bodyw Mov Ther. 2022 Oct;32:1-6. doi: 10.1016/j.jbmt.2022.05.017. Epub 2022 May 19. PMID: 36180134.

Wiegand S 2015	49	10.7556/jaoa.2015.019	Wiegand S, Bianchi W, Quinn TA, Best M, Fotopoulos T	Osteopathic manipulative treatment for self-reported fatigue, stress, and depression in first-year osteopathic medical students	J Am Osteopath Assoc. 2015 Feb;115(2):84-93. doi: 10.7556/jaoa.2015.019. PMID: 25637614.
Terrell ZT 2022	50	10.1515/jom-2021-0203	Terrell ZT, Moudy SC, Hensel KL, Patterson RM	Effects of osteopathic manipulative treatment vs. osteopathic cranial manipulative medicine on Parkinsonian gait	J Osteopath Med. 2022 Feb 14;122(5):243-251. doi: 10.1515/jom-2021-0203. PMID: 35148036.
Detoni R 2022	51	10.1016/j.jbmt.2021.09.021	Detoni R, Hartz CS, Fusatto EL, Bicalho E, Nacimento-Moraes KSG, Rizzatti-Barbosa CM, Lopes FOT	Relationship between osteopathic manipulative treatment of the temporomandibular joint, molar shim and the orthostatic position: A randomized, controlled and double blinded study	J Bodyw Mov Ther. 2022 Jan;29:187-197. doi: 10.1016/j.jbmt.2021.09.021. Epub 2021 Oct 22. PMID: 35248270.
Arienti C 2011	52	10.1038/sc.2010.170	Arienti C, Daccò S, Piccolo I, Redaelli T	Osteopathic manipulative treatment is effective on pain control associated to spinal cord injury	Spinal Cord. 2011 Apr;49(4):515-9. doi: 10.1038/sc.2010.170. Epub 2010 Dec 7. PMID: 21135862.
Yosri MM 2022	53	10.1515/jom-2021-0255	Yosri MM, Hamada HA, Yousef AM	Effect of visceral manipulation on menstrual complaints in women with polycystic ovarian syndrome	J Osteopath Med. 2022 May 2;122(8):411-422. doi: 10.1515/jom-2021-0255. PMID: 35488711.

Fraix M 2021	54	10.7556/jaoa.2020.147	Fraix M, Badran S, Graham V, Redman-Bentley D, Hurwitz EL, Quan VL, Yim M, Hudson-McKinney M, Seffinger MA	Osteopathic manipulative treatment in individuals with vertigo and somatic dysfunction: a randomized, controlled, comparative feasibility study	J Osteopath Med. 2021 Jan 1;121(1):71-83. doi: 10.7556/jaoa.2020.147. PMID: 33125033.
Noto Bell L 2019	55	10.7556/jaoa.2019.100	Noto-Bell L, Vogel BN, Senn DE	Effects of Post-Isometric Relaxation on Ankle Plantarflexion and Timed Flutter Kick in Pediatric Competitive Swimmers	J Am Osteopath Assoc. 2019 Sep 1;119(9):569-577. doi: 10.7556/jaoa.2019.100. PMID: 31449303.
Manzotti A 2020 B	56	10.3390/ijerph17093250	Manzotti A, Viganoni C, Lauritano D, Bernasconi S, Paparo A, Risso R, Nanussi A	Evaluation of the Stomatognathic System before and after Osteopathic Manipulative Treatment in 120 Healthy People by Using Surface Electromyography	Int J Environ Res Public Health. 2020 May 7;17(9):3250. doi: 10.3390/ijerph17093250. PMID: 32392700; PMCID: PMC7246931.
Wojcik M 2022	57	10.31083/j.jomh1806140	Wójcik M, Siatkowski I, Żekanowska E	A Proposal for the Use of Craniosacral Therapy in Firefighter Cadets to Decrease Cortisol Levels and Improve Postural Stability. A Randomized Trial	NA
Abdelfattah ASI 2019	58	10.4328/ACAM.6045	Abdelfattah, ASI, Raoof NALA, Nasef SUS, Ali RR, Swailm RS	Effect of selected osteopathic lymphatic techniques on	NA

				immune system in healthy subjects: A randomized control trial	
Deodato M 2019	59	10.7556/jaoa.2019.093	Deodato M, Guolo F, Monticco A, Fornari M, Manganotti P, Granato A	Osteopathic Manipulative Therapy in Patients With Chronic Tension-Type Headache: A Pilot Study	J Am Osteopath Assoc. 2019 Aug 12. doi: 10.7556/jaoa.2019.093. Epub ahead of print. PMID: 31404469.
Lagrange A 2019	60	10.1016/j.ejogrb.2019.08.003	Lagrange A, Decoux D, Briot N, Hennequin A, Coudert B, Desmoulins I, Bertaut A	Visceral osteopathic manipulative treatment reduces patient reported digestive toxicities induced by adjuvant chemotherapy in breast cancer: A randomized controlled clinical study	Eur J Obstet Gynecol Reprod Biol. 2019 Oct;241:49-55. doi: 10.1016/j.ejogrb.2019.08.003. Epub 2019 Aug 12. PMID: 31430616
Papa L 2017	61	10.1016/j.jbmt.2017.03.001	Papa L, Amodio A, Biffi F, Mandara A	Impact of osteopathic therapy on proprioceptive balance and quality of life in patients with dizziness	J Bodyw Mov Ther. 2017 Oct;21(4):866-872. doi: 10.1016/j.jbmt.2017.03.001. Epub 2017 Mar 6. PMID: 29037641.
Cases Sole R 2022	62	10.3389/fnbeh.2022.860223	Cases-Solé R, Varillas-Delgado D, Astals-Vizcaino M, García-Algar Ó	Efficacy and Feasibility of an Osteopathic Intervention for Neurocognitive and Behavioral Symptoms Usually Associated With Fetal Alcohol Spectrum Disorder	Front Behav Neurosci. 2022 Mar 15;16:860223. doi: 10.3389/fnbeh.2022.860223. PMID: 35368309; PMCID: PMC8965441.
Santiago RJ 2022	63	10.1016/j.ctcp.2021.101507	Santiago RJ, Esteves JE, Baptista JS,	Results of a feasibility randomised	Complement Ther Clin Pract. 2022 Feb;46:101507. doi: 10.1016/j.ctcp.2021.101507.

			Magalhães A, Costa JT	controlled trial of osteopathy on neck-shoulder pain in computer users	Epub 2021 Nov 3. PMID: 34753085
Cerritelli F 2015 B	64	10.1016/j.ctim.2015.01.011	Cerritelli F, Ginevri L, Messi G, Caprari E, Di Vincenzo M, Renzetti C, Cozzolino V, Barlafante G, Foschi N, Provinciali L	Clinical effectiveness of osteopathic treatment in chronic migraine: 3-Armed randomized controlled trial	Complement Ther Med. 2015 Apr;23(2):149-56. doi: 10.1016/j.ctim.2015.01.011. Epub 2015 Jan 21. PMID: 25847552.
Zarucchi A 2020	65	10.3233/NRE-203068	Zarucchi A, Vismara L, Frazzitta G, Mauro A, Priano L, Maestri R, Bergna A, Tarantino AG	Efficacy of Osteopathic Manipulative Treatment on postural control in Parkinsonian patients with Pisa syndrome: A pilot randomized placebo-controlled trial	NeuroRehabilitation. 2020;46(4):529-537. doi: 10.3233/NRE-203068. PMID: 32538880.
Bagagiolo D 2022	66	10.1055/s-0042-1758723	Bagagiolo D, Priolo CG, Favre EM, Pangallo A, Didio A, Sbarbaro M, Borro T, Daccò S, Manzoni P, Farina D	A Randomized Controlled Trial of Osteopathic Manipulative Therapy to Reduce Cranial Asymmetries in Young Infants with Nonsynostotic Plagiocephaly	Am J Perinatol. 2022 Dec;39(S 01):S52-S62. doi: 10.1055/s-0042-1758723. Epub 2022 Nov 30. PMID: 36451623.
Danielo Jouhier M 2021	67	10.1136/archdischild-2020-319219	Danielo Jouhier M, Boscher C, Roze JC, Cailleau N, Chaligne F, Legrand A, Flamant C, Muller JB	Osteopathic manipulative treatment to improve exclusive breast feeding at 1 month	Arch Dis Child Fetal Neonatal Ed. 2021 Nov;106(6):591-595. doi: 10.1136/archdischild-2020-319219. Epub 2021 Mar 31. PMID: 33789971.
Licciardone JC 2013 A	68	10.1370/afm.1468	Licciardone JC, Minotti DE, Gatchel	Osteopathic manual treatment and ultrasound	Ann Fam Med. 2013 Mar-Apr;11(2):122-9. doi: 10.1370/afm.1468. PMID:

			RJ, Kearns CM, Singh KP	therapy for chronic low back pain: a randomized controlled trial	23508598; PMCID: PMC3601389.
Dugailly P-M 2014	69	10.1016/j.ijosm.2013.08.001	Dugailly PM, Fassin SB, Maroye L, Evers L, Klein P, Feipel VR	Effect of a general osteopathic treatment on body satisfaction, global self perception and anxiety: A randomized trial in asymptomatic female students	NA
Accorsi A 2014 B	70	10.7556/jaoa.2014.074	Accorsi A, Lucci C, Di Mattia L, Granchelli C, Barlafante G, Fini F, Pizzolorusso G, Cerritelli F, Pincherle M	Effect of Osteopathic Manipulative Therapy in the Attentive Performance of Children With Attention-Deficit/Hyperactivity Disorder	J Am Osteopath Assoc. 2014 May;114(5):374-81. doi: 10.7556/jaoa.2014.074. PMID: 24778002.
Yao SC 2020	71	10.7556/jaoa.2020.099	Yao SC, Zwibel H, Angelo N, Leder A, Mancini J	Effectiveness of Osteopathic Manipulative Medicine vs Concussion Education in Treating Student Athletes With Acute Concussion Symptoms	J Am Osteopath Assoc. 2020 Aug 7. doi: 10.7556/jaoa.2020.099. Epub ahead of print. PMID: 32766808.
Minarini G 2018	72	10.1016/j.ijosm.2018.10.002	Minarini G, Ford M, Esteves J	Immediate effect of T2, T5, T11 thoracic spine manipulation of asymptomatic patient on autonomic nervous system response: Single-blind, parallel-arm	NA

				controlled-group experiment	
Vismara L 2012	73	10.1016/j.math.2012.05.002	Vismara L, Cimolin V, Menegoni F, Zaina F, Galli M, Negrini S, Villa V, Capodaglio P	Osteopathic manipulative treatment in obese patients with chronic low back pain: a pilot study	Man Ther. 2012 Oct;17(5):451-5. doi: 10.1016/j.math.2012.05.002. Epub 2012 May 31. PMID: 22658268.
Cerritelli F 2020	74	10.1038/s41598-020-60253-6	Cerritelli F, Chiacchiaretta P, Gambi F, Perrucci MG, Barassi G, Visciano C, Bellomo RG, Saggini R, Ferretti A	Effect of manual approaches with osteopathic modality on brain correlates of interoception: an fMRI study	Sci Rep. 2020 Feb 21;10(1):3214. doi: 10.1038/s41598-020-60253-6. PMID: 32081945; PMCID: PMC7035282.
Tramontano M 2020 B	75	10.7556/jaoa.2020.029	Tramontano M, Pagnotta S, Lunghi C, Manzo C, Manzo F, Consolo S, Manzo V	Assessment and Management of Somatic Dysfunctions in Patients With Patellofemoral Pain Syndrome	J Am Osteopath Assoc. 2020 Mar 1;120(3):165-173. doi: 10.7556/jaoa.2020.029. PMID: 32091560.
Ghasemi C 2020	76	10.35975/apic.v24i5.1362	Ghasemi C, Amiri A, Sarrafzadeh J, Dadgoo M, Maroufi N	Comparison of the effects of craniosacral therapy, muscle energy technique, and sensorimotor training on non specific chronic low back pain	NA
Arienti C 2020	77	10.1016/j.jbmt.2020.07.002	Arienti C, Farinola F, Ratti S, Daccò S, Fasulo L	Variations of HRV and skin conductance reveal the influence of CV4 and Rib Raising techniques on autonomic balance: A randomized controlled clinical trial	J Bodyw Mov Ther. 2020 Oct;24(4):395-401. doi: 10.1016/j.jbmt.2020.07.002. Epub 2020 Jul 31. PMID: 33218540.

Schwerla F 2014	78	10.1016/j.ijosm.2014.04.003	Schwerla F, Wirthwein P, Rutz M, Resch	Osteopathic treatment in patients with primary dysmenorrhoea: A randomised controlled trial	NA
Iqbal M 2020	79	10.5455/JPMA.23971	Iqbal M, Riaz H, Ghous M, Masood K	Comparison of Spencer muscle energy technique and Passive stretching in adhesive capsulitis: A single blind randomized control trial	J Pak Med Assoc. 2020 Dec;70(12(A)):2113-2118. doi: 10.5455/JPMA.23971. PMID: 33475581.
Licciardone JC 2013 B	80	10.7556/jaoa.2013.043	Licciardone JC, Aryal S	Prevention of progressive back-specific dysfunction during pregnancy: an assessment of osteopathic manual treatment based on Cochrane Back Review Group criteria	J Am Osteopath Assoc. 2013 Oct;113(10):728-36. doi: 10.7556/jaoa.2013.043. PMID: 24084800.
Galindez Ibarbengoetxea X 2017	81	10.1016/j.ijosm.2016.11.004	Galindez-Ibarbengoetxea X, Setuain I, Gonzales-Izal M, Jauregi A, Ramirez-Velez R, Andersen LL, Izquierd M	Randomised controlled pilot trial of high-velocity, low-amplitude manipulation on cervical and upper thoracic spine levels in asymptomatic subjects	NA
Espi Lopez GV 2018	82	10.1097/MD.0000000000013811	Espí-López GV, Inglés M, Soliva-Cazabán I, Serra-Añó P	Effect of the soft-tissue techniques in the quality of life in patients with Crohn's disease: A randomized controlled trial	Medicine (Baltimore). 2018 Dec;97(51):e13811. doi: 10.1097/MD.0000000000013811. PMID: 30572544; PMCID: PMC6320155.

Auger K 2021	83	10.1515/jom-2020-0132	Auger K, Shedlock G, Coutinho K, Myers NE, Lorenzo S	Effects of osteopathic manipulative treatment and bio-electromagnetic energy regulation therapy on lower back pain	J Osteopath Med. 2021 Mar 2;121(6):561-569. doi: 10.1515/jom-2020-0132. PMID: 33694338.
Swender DA 2014	84	10.7556/jaoa.2014.095	Swender DA, Thompson G, Schneider K, McCoy K, Patel A	Osteopathic manipulative treatment for inpatients with pulmonary exacerbations of cystic fibrosis: effects on spirometry findings and patient assessments of breathing, anxiety, and pain	J Am Osteopath Assoc. 2014 Jun;114(6):450-8. doi: 10.7556/jaoa.2014.095. PMID: 24917632.
Snider KT 2018	85	10.7556/jaoa.2018.035	Snider KT, Redman CL, Edwards CR, Bhatia S, Kondrashova T	Ultrasonographic Evaluation of the Effect of Osteopathic Manipulative Treatment on Sacral Base Asymmetry	J Am Osteopath Assoc. 2018 Mar 1;118(3):159-169. doi: 10.7556/jaoa.2018.035. PMID: 29480916.
Jones LM 2021	86	10.1515/jom-2020-0040	Jones LM, Regan C, Wolf K, Bryant J, Rakowsky A, Pe M, Snyder DA	Effect of osteopathic manipulative treatment on pulmonary function testing in children with asthma	J Osteopath Med. 2021 May 7;121(6):589-596. doi: 10.1515/jom-2020-0040. PMID: 33962511.
Zanotti E 2012	87	10.1016/j.ctim.2011.10.008	Zanotti E, Berardinelli P, Bizzarri C, Civardi A, Manstretta A, Rossetti S, Fracchia C.	Osteopathic manipulative treatment effectiveness in severe chronic obstructive pulmonary disease: a pilot study	Complement Ther Med. 2012 Feb-Apr;20(1-2):16-22. doi: 10.1016/j.ctim.2011.10.008. Epub 2011 Nov 27. PMID: 22305244.

Saggio G 2011	88		Saggio G, Docimo S, Pilc J, Norton J, Gilliar W.	Impact of osteopathic manipulative treatment on secretory immunoglobulin a levels in a stressed population	J Am Osteopath Assoc. 2011 Mar;111(3):143-7. PMID: 21464262.
Bruscemi A 2019 A	89	10.1515/jcim-2018-0128	Buscemi A, Pennisi V, Rapisarda A, Pennisi A, Coco M	Efficacy of osteopathic treatment in patients with stable moderate-to-severe chronic obstructive pulmonary disease: a randomized controlled pilot study	J Complement Integr Med. 2019 Aug 23;17(1):/j/jcim.2019.17.issue-1/jcim-2018-0128/jcim-2018-0128.xml. doi: 10.1515/jcim-2018-0128. PMID: 31442204.
Steele KM 2014	90	10.7556/jaoa.2014.094	Steele KM, Carreiro JE, Viola JH, Conte JA, Ridpath LC.	Effect of osteopathic manipulative treatment on middle ear effusion following acute otitis media in young children: a pilot study	J Am Osteopath Assoc. 2014 Jun;114(6):436-47. doi: 10.7556/jaoa.2014.094. PMID: 24917631.
Wieting JM 2013	91		Wieting JM, Beal C, Roth GL, Gorbis S, Dillard L, Gilliland D, Rowan J.	The effect of osteopathic manipulative treatment on postoperative medical and functional recovery of coronary artery bypass graft patients	J Am Osteopath Assoc. 2013 May;113(5):384-93. PMID: 23667192.
Hubert D 2014	92	10.1371/journal.pone.0102465	Hubert D, Soubeiran L, Gourmelon F, Grenet D, Serreau R, Perrodeau E, Zegarra-Parodi R, Boutron I	Impact of osteopathic treatment on pain in adult patients with cystic fibrosis--a pilot randomized	PLoS One. 2014 Jul 16;9(7):e102465. doi: 10.1371/journal.pone.0102465. PMID: 25029347; PMCID: PMC4100932

				controlled study	
Gesslbauer C 2018	93	10.1080/09638288.2016.1269368	Gesslbauer C, Vavti N, Keilani M, Mickel M, Crevenna R.	Effectiveness of osteopathic manipulative treatment versus osteopathy in the cranial field in temporomandibular disorders - a pilot study	Disabil Rehabil. 2018 Mar;40(6):631-636. doi: 10.1080/09638288.2016.1269368. Epub 2016 Dec 28. PMID: 28029069.
Cerritelli F 2021	94	10.1038/s41598-021-83893-8	Cerritelli F, Chiacchiaretta P, Gambi F, Saggini R, Perrucci MG, Ferretti A.	Osteopathy modulates brain-heart interaction in chronic pain patients: an ASL study	Sci Rep. 2021 Feb 25;11(1):4556. doi: 10.1038/s41598-021-83893-8. PMID: 33633195; PMCID: PMC7907192.
Curtis P 2011	95	10.1089/acm.2010.0277	Curtis P, Gaylord SA, Park J, Faurot KR, Coble R, Suchindran C, Coeytaux RR, Wilkinson L, Mann JD.	Credibility of low-strength static magnet therapy as an attention control intervention for a randomized controlled study of CranioSacral therapy for migraine headaches	J Altern Complement Med. 2011 Aug;17(8):711-21. doi: 10.1089/acm.2010.0277. Epub 2011 Jul 6. PMID: 21732734; PMCID: PMC3142630.
Naci B 2022	96	10.1016/j.amjcard.2021.09.023	Naci B, Demir R, Onder OO, Sinan UY, Kucukoglu MS.	Effects of Adding Respiratory Training to Osteopathic Manipulative Treatment on Exhaled Nitric Oxide Level and Cardiopulmonary Function in Patients With Pulmonary Arterial Hypertension	Am J Cardiol. 2022 Jan 1;162:184-190. doi: 10.1016/j.amjcard.2021.09.023. Epub 2021 Oct 24. PMID: 34706819.
Da Silva RCV 2013	97	10.1111/j.1442-2050.2012.01372.x	da Silva RC, de Sá CC,	Increase of lower	Dis Esophagus. 2013 Jul;26(5):451-6. doi:

			Pascual-Vaca ÁO, de Souza Fontes LH, Herbella Fernandes FA, Dib RA, Blanco CR, Queiroz RA, Navarro-Rodriguez T.	esophageal sphincter pressure after osteopathic intervention on the diaphragm in patients with gastroesophageal reflux	10.1111/j.1442-2050.2012.01372.x. Epub 2012 Jun 7. PMID: 22676647.
Buscemi A 2019 B	98	10.3892/etm.2019.7628	Buscemi A, Petralia MC, Ramaci T, Rapisarda A, Provazza C, Di Corrado D, Perciavalle V, Perciavalle V, Coco M.	Ergojump evaluation of the explosive strength in volleyball athletes pre- and post-fascial treatment	Exp Ther Med. 2019 Aug;18(2):1470-1476. doi: 10.3892/etm.2019.7628. Epub 2019 May 29. PMID: 31384337; PMCID: PMC6639914.
Mataran Penaroccha GA 2011	99	10.1093/ecam/nep125	Matarán-Peñarrocha GA, Castro-Sánchez AM, García GC, Moreno-Lorenzo C, Carreño TP, Zafra MD.	Influence of craniosacral therapy on anxiety, depression and quality of life in patients with fibromyalgia	Evid Based Complement Alternat Med. 2011;2011:178769. doi: 10.1093/ecam/nep125. Epub 2011 Jun 15. PMID: 19729492; PMCID: PMC3135864.
Hensel KL 2015	100	10.1016/j.ajog.2014.07.043	Hensel KL, Buchanan S, Brown SK, Rodriguez M, Cruser dA.	Pregnancy Research on Osteopathic Manipulation Optimizing Treatment Effects: the PROMOTE study	Am J Obstet Gynecol. 2015 Jan;212(1):108.e1-9. doi: 10.1016/j.ajog.2014.07.043. Epub 2014 Jul 25. PMID: 25068560; PMCID: PMC4275366.
Abenavoli A 2020	101	10.1016/j.jbmt.2020.07.017	Abenavoli A, Badi F, Barbieri M, Bianchi M, Biglione G, Dealessi C, Grandini M, Lavazza C, Mapelli L, Milano V, Monti L, Seppia S, Tresoldi M, Maggiani A.	Cranial osteopathic treatment and stress-related effects on autonomic nervous system measured by salivary markers: A pilot study	J Bodyw Mov Ther. 2020 Oct;24(4):215-221. doi: 10.1016/j.jbmt.2020.07.017. Epub 2020 Aug 4. PMID: 33218514.

Silva ACO 2018	102	10.1155/2018/4929271	Silva ACO, Biasotto-Gonzalez DA, Oliveira FHM, Andrade AO, Gomes CAFP, Lanza FC, Amorim CF, Politti F.	Effect of Osteopathic Visceral Manipulation on Pain, Cervical Range of Motion, and Upper Trapezius Muscle Activity in Patients with Chronic Nonspecific Neck Pain and Functional Dyspepsia: A Randomized, Double-Blind, Placebo-Controlled Pilot Study	Evid Based Complement Alternat Med. 2018 Nov 11;2018:4929271. doi: 10.1155/2018/4929271. PMID: 30534176; PMCID: PMC6252226.
Esterov D 2021	103	10.1515/jom-2020-0035	Esterov D, Thomas A, Weiss K	Osteopathic manipulative medicine in the management of headaches associated with postconcussion syndrome	J Osteopath Med. 2021 Apr 9;121(7):651-656. doi: 10.1515/jom-2020-0035. PMID: 33831981.
Giles PD 2013	104	10.1089/acm.2011.0031	Giles PD, Hensel KL, Pacchia CF, Smith ML.	Suboccipital decompression enhances heart rate variability indices of cardiac control in healthy subjects	J Altern Complement Med. 2013 Feb;19(2):92-6. doi: 10.1089/acm.2011.0031. Epub 2012 Sep 20. PMID: 22994907; PMCID: PMC3576914.
Noll DR 2013	105	10.7556/jaoa.2013.003	Noll DR.	The short-term effect of a lymphatic pump protocol on blood cell counts in nursing home residents with limited mobility: a pilot study	J Am Osteopath Assoc. 2013 Jul;113(7):520-8. doi: 10.7556/jaoa.2013.003. Erratum in: J Am Osteopath Assoc. 2013 Sep;113(9):662. PMID: 23843375.
Pasin Neto H 2020	106	10.7759/cureus.8058	Pasin Neto H, Borges RA.	Visceral Mobilization and Functional Constipation in	Cureus. 2020 May 11;12(5):e8058. doi: 10.7759/cureus.8058. PMID:

				Stroke Survivors: A Randomized, Controlled, Double-Blind, Clinical Trial	32537276; PMCID: PMC7286593.
Bialoszewski D 2014	107	10.5604/15093492.1135120	Białoszewski D, Bebelski M, Lewandowska M, Słupik A.	Utility of craniosacral therapy in treatment of patients with non-specific low back pain. Preliminary report	Ortop Traumatol Rehabil. 2014 Nov-Dec;16(6):605-15. doi: 10.5604/15093492.1135120. PMID: 25694375.
Fornari M 2017	108	10.7556/jaoa.2017.110	Fornari M, Carnevali L, Sgoifo A.	Single Osteopathic Manipulative Therapy Session Dampens Acute Autonomic and Neuroendocrine Responses to Mental Stress in Healthy Male Participants	J Am Osteopath Assoc. 2017 Sep 1;117(9):559-567. doi: 10.7556/jaoa.2017.110. PMID: 28846122.
Stepnik J 2020	109	10.1371/journal.pone.0235308	Stępnik J, Kędra A, Czaprowski D.	Short-term effect of osteopathic manual techniques (OMT) on respiratory function in healthy individuals	PLoS One. 2020 Jun 30;15(6):e0235308. doi: 10.1371/journal.pone.0235308. PMID: 32603336; PMCID: PMC7326176.
Shi X 2011	110		Shi X, Rehrer S, Prajapati P, Stoll ST, Gamber RG, Downey HF.	Effect of cranial osteopathic manipulative medicine on cerebral tissue oxygenation	J Am Osteopath Assoc. 2011 Dec;111(12):660-6. PMID: 22182951.
Klein R 2013	111	10.1016/j.ctim.2012.11.003	Klein R, Bareis A, Schneider A, Linde K.	Strain-counterstrain to treat restrictions of the mobility of the cervical spine in patients with neck pain: a	Complement Ther Med. 2013 Feb;21(1):1-7. doi: 10.1016/j.ctim.2012.11.003. Epub 2012 Dec 7. PMID: 23374199.

				sham-controlled randomized trial	
Marske C 2018	112	10.1089/acm.2017.0178	Marske C, Bernard N, Palacios A, Wheeler C, Preiss B, Brown M, Bhattacharya S, Klapstein G.	Fibromyalgia with Gabapentin and Osteopathic Manipulative Medicine: A Pilot Study	J Altern Complement Med. 2018 Apr;24(4):395-402. doi: 10.1089/acm.2017.0178. Epub 2018 Jan 3. PMID: 29298077.
Tozzi P 2012	113	10.1016/j.jbmt.2012.02.001	Tozzi P, Bongiorno D, Vitturini C.	Low back pain and kidney mobility: local osteopathic fascial manipulation decreases pain perception and improves renal mobility	J Bodyw Mov Ther. 2012 Jul;16(3):381-391. doi: 10.1016/j.jbmt.2012.02.001. Epub 2012 Mar 3. PMID: 22703751.
Moustafa IM 2015	114	10.1007/s00296-015-3248-7	Moustafa IM, Diab AA	The addition of upper cervical manipulative therapy in the treatment of patients with fibromyalgia: a randomized controlled trial	Rheumatol Int. 2015 Jul;35(7):1163-74. doi: 10.1007/s00296-015-3248-7. Epub 2015 Mar 18. PMID: 25782585.
Herzhaft Le Roy J 2017	115	10.1177/0890334416679620	Herzhaft-Le Roy J, Xhignesse M, Gaboury I.	Efficacy of an Osteopathic Treatment Coupled With Lactation Consultations for Infants' Biomechanical Sucking Difficulties	J Hum Lact. 2017 Feb;33(1):165-172. doi: 10.1177/0890334416679620. Epub 2016 Dec 27. PMID: 28027445.
Wojcik M 2019	116	10.1016/j.ijosm.2019.04.007	Małgorzata Wójcika, Inga Dziembowskab, Paweł Izdebskid, Ewa Zekanowska.	Pilot randomized single-blind clinical trial, craniosacral therapy vs control on physiological reaction to	NA

				math task in male athletes	
Jardine WM 2012	117	10.1016/j.ijosm.2012.07.001	Wendy M. Jardine, Carol Gillis, Derek Rutherford.	The effect of osteopathic manual therapy on the vascular supply to the lower extremity in individuals with knee osteoarthritis: A randomized trial	NA
Noccioli G 2014	118	10.11138/GIOG/2014.36.2.339	G. Noccioli, N. Noccioli, S. Graziosi, M. Petracca, O. Gambardella, F. Dammando.	Randomized clinical trial on the effects of osteopathic treatment in menopausal women	NA
De Oliveira Meirelles F 2020	119	10.3233/BMR-181355	de Oliveira Meirelles F, de Oliveira Muniz Cunha JC, da Silva EB.	Osteopathic manipulation treatment versus therapeutic exercises in patients with chronic nonspecific low back pain: A randomized, controlled and double-blind study	J Back Musculoskelet Rehabil. 2020;33(3):367-377. doi: 10.3233/BMR-181355. PMID: 31658037.
Hidalgo B 2018	120	10.3233/BMR-170963	Hidalgo B, Hall T, Berwart M, Biernaux E, Detrembleur C.	The immediate effects of two manual therapy techniques on ankle musculoarticular stiffness and dorsiflexion range of motion in people with chronic ankle rigidity: A randomized clinical trial	J Back Musculoskelet Rehabil. 2018;31(3):515-524. doi: 10.3233/BMR-170963. PMID: 29309040.
Romagnoli M 2021	121	10.23736/S0393-3660.20.04305-3	Marco Romagnoli, Andrea Tarantino,	Modulation of vestibulo-ocular reflex gain-symmetry	NA

			Matteo Galli, Daniele Origo	induced by osteopathic manipulations on the fascial system in young healthy subjects: a three-arm randomized controlled trial	
Mazreati N 2021	122	10.18502/npt.v8i4.6707	Nasim Mazreati, Zahra Rahemi, Mohammad Aghajani, Neda Mirbagher Ajorpaz, Elaheh Mianehsaz	Effect of craniosacral therapy on the intensity of chronic back pain of nurses: A randomized controlled trial	NA
Huard Y 2013	123		Yannick Huard	Gait disturbance in the elderly: Contribution of an osteopathic treatment	NA
Ghasemi C 2021	124	10.35975/apic.v25i2.1458	Cobra Ghasemi, Ali Amiri, Javad Sarrafzadeh, Mehdi Dadgoo	Effects of craniosacral therapy and sensorimotor training on pain, disability, depression and quality of life of patients with nonspecific chronic low back pain: a randomized clinical trial	NA
Galindez Ibarbengoetxea X 2018	125		Galindez-Ibarbengoetxea X, Setuain I, Ramírez-Velez R, Andersen LL, González-Izal M, Jauregi A, Izquierdo M	Immediate Effects of Osteopathic Treatment Versus Therapeutic Exercise on Patients With Chronic Cervical Pain	Altern Ther Health Med. 2018 May;24(3):24-32. PMID: 29135458.

Brandl A 2023	126	10.3390/jcm12041248	Brandl A, Egner C, Reer R, Schmidt T, Schleip R.	Immediate Effects of Myofascial Release Treatment on Lumbar Microcirculation: A Randomized, Placebo-Controlled Trial	J Clin Med. 2023 Feb 4;12(4):1248. doi: 10.3390/jcm12041248. PMID: 36835784; PMCID: PMC9959802.
Wójcik M 2023	127	10.3390/bs13110914	Wójcik M, Bordoni B, Siatkowski I, Żekanowska E.	The Effect of Craniosacral Therapy on Blood Levels of Stress Hormones in Male Firefighter Cadets: A Randomized Clinical Trial	Behav Sci (Basel). 2023 Nov 8;13(11):914. doi: 10.3390/bs13110914. PMID: 37998661; PMCID: PMC10669461.
Ożóg P 2023	128	10.3390/ijerph20032198	Ożóg P, Weber-Rajek M, Radzimińska A, Goch A.	Analysis of Postural Stability Following the Application of Myofascial Release Techniques for Low Back Pain-A Randomized-Controlled Trial	Int J Environ Res Public Health. 2023 Jan 26;20(3):2198. doi: 10.3390/ijerph20032198. PMID: 36767565; PMCID: PMC9915703.
Martínez-Lentisco MDM 2023	129	10.3390/healthcare11182600	Martínez-Lentisco MDM, Martín-González M, García-Torrecillas JM, Antequera-Soler E, Chillón-Martínez R.	Osteopathic Manual Therapy for Infant Colic: A Randomised Clinical Trial	Healthcare (Basel). 2023 Sep 21;11(18):2600. doi: 10.3390/healthcare11182600. PMID: 37761797; PMCID: PMC10531355.
Boas Fernandes 2023	130	10.1016/j.jbmt.2023.04.006	Boas Fernandes WV, Politti F, Blanco CR, Garcia Lucareli PR, Gomes CAFP,	Effect of osteopathic visceral manipulation for individuals with functional constipation	J Bodyw Mov Ther. 2023 Apr;34:96-103. doi: 10.1016/j.jbmt.2023.04.006. Epub 2023 Apr 6. PMID: 37301564.

			Corrêa FI, Corrêa JCF.	and chronic nonspecific low back pain: Randomized controlled trial	
Bohlen L 2022	13 1	10.1038/s41598-022-20452-9	Bohlen L, Schwarze J, Richter J, Gietl B, Lazarov C, Kopyakova A, Brandl A, Schmidt T.	Effect of osteopathic techniques on human resting muscle tone in healthy subjects using myotonometry: a factorial randomized trial	Sci Rep. 2022 Oct 10;12(1):16953. doi: 10.1038/s41598-022-20452-9. PMID: 36217012; PMCID: PMC9551048.

Supplementary Table S3. Characteristics of the journals included in the selection (according to In-Cites Journal Citation Reports and Science Direct). See the page below.

Journal	Journal Impact Factor	5-Year Impact Factor	Journal quartile	Year	Publication Option
JAMA Internal Medicine	22,5	22,5	Q1	2023	Hybrid
American Journal of Obstetrics and Gynecology	8,7	8.8	Q1	2023	Hybrid
BMC Complementary and Alternative Medicine	4,7	4,4	Q1	2021	Open access
International Journal of Environmental Research and Public Health	4,6	4,8	Q2	2021	Open access
Archives of Disease in Childhood	4,3	3,9	Q1	2023	Hybrid
Scientific Reports	3,8	4,3	Q1	2023	Open access
Clinical and Experimental Dermatology	3,7	3,4	Q1	2023	Hybrid
The Annals Of Thoracic Surgery	3,6	3,8	Q1	2023	Hybrid
Archives of Physical Medicine and Rehabilitation	3,6	3,9	Q1	2023	Hybrid
Acta Obstetricia et Gynecologica Scandinavica	3,5	4	Q1	2023	Open access
Therapeutic Advances in Musculoskeletal Disease	3,4	3,7	Q2	2023	Open access
Complementary therapies in medicine	3,3	3,3	Q1	2023	Open access
Frontiers in Physiology	3,2	4	Q2	2023	Open access
Rheumatology International	3,2	2,9	Q2	2023	Hybrid
Life	3,2	3,1	Q1	2023	Open access
Journal of Clinical Medicine	3	3,4	Q1	2023	Open access
PLOS ONE	2,9	3,3	Q1	2023	Open access
The Clinical Journal of Pain	2,9	3,8	Q1	2023	Hybrid

Brain Sciences	2,7	3	Q3	2023	Open access
Clinical Rehabilitation	2,6	3,4	Q1	2023	Hybrid
Frontiers in Behavioral Neuroscience	2,6	3,2	Q2	2023	Open access
Evidence-Based Complementary and Alternative Medicine	2,6	3	Q3	2021	Open access
Manual Therapy	2,6	2,8	Q1	2018	Hybrid
Behavioral Sciences	2,5	2,7	Q2	2023	Open access
Healthcare	2,4	2,5	Q2	2023	Open access
Experimental and Therapeutic Medicine	2,4	2,3	Q3	2023	Hybrid
The American Journal of Cardiology	2,3	2,3	Q1	2023	Hybrid
Diseases of the Esophagus	2,3	2,7	Q3	2023	Hybrid
Journal of Alternative and Complementary Medicine	2,3	3,7	Q2	2023	Hybrid
Annals of Family Medicine	2,2	4,8	Q1	2023	Open access
Complementary Therapies in Clinical Practice	2,2	2,8	Q2	2023	Hybrid
Journal of Human Lactation	2,1	2,5	Q2	2023	Hybrid
European Journal of Obstetrics & Gynecology and Reproductive Biology	2,1	2,3	Q2	2023	Hybrid
Spinal Cord	2,1	2,6	Q1	2023	Hybrid
Disability and Rehabilitation	2,1	2,5	Q1	2023	Hybrid
BMC Pediatrics	2	2,4	Q2	2023	Open access
Alternative Therapies in Health and Medicine	1,9	1,7	Q3	2023	Hybrid
Journal of Complementary and Integrative Medicine	1,8	NA	Q2	2023	Hybrid
NeuroRehabilitation	1,7	2,1	Q2	2023	Hybrid
Journal of Manual and Manipulative Therapy	1,6	1,6	Q2	2023	Hybrid
American Journal of Perinatology	1,5	1,7	Q2	2023	Hybrid
Journal of Back and Musculoskeletal Rehabilitation	1,4	1,5	Q3	2023	Hybrid
Journal of Osteopathic Medicine	1,4	1,4	Q2	2023	Open access
Medicine	1,3	1,6	Q2	2023	Open access
Turkish Society of Physical Medicine and Rehabilitation	1,3	1,4	Q2	2023	Open access
Journal of Bodywork & Movement Therapies	1,2	1,6	Q3	2023	Hybrid
Journal of Manipulative and Physiological Therapeutics	1,2	1,5	Q3	2023	Hybrid
International Journal of Osteopathic Medicine	1,1	1,6	Q3	2023	Hybrid

Complementary Medicine Research	1,1	1,3	Q3	2023	Hybrid
Cureus	1	1,1	Q3	2023	Open access
Journal of Chiropractic Medicine	0,8	1,3	Q4	2023	Hybrid
Journal of the Pakistan Medical Association	0,8	1	Q4	2023	Hybrid
Nursing Practice Today	0,7	NA	Q4	2023	Open access
Journal of Men's Health	0,6	NA	Q4	2023	Open access
Ortopedia Traumatologia Rehabilitacja	0,5	NA	Q3	2022	Hybrid
Anaesthesia, Pain & Intensive Care	0,2	NA	Q4	2022	Open access
Clinical Cases in Mineral and Bone Metabolism	0,1	NA	Q4	2020	Open access
The Annals of Clinical and Analytical Medicine	0,1	0,1	Q4	2023	Open access
Giornale Italiano di Ostetricia e Ginecologia	0,02	NA	Q4	2020	Hybrid
The American Academy of Osteopathy Journal	0	NA	Q4	2022	Hybrid
Gazzetta Medica Italiana Archivio per le Scienze Mediche	NA	NA	Q4	2023	Hybrid
